# Inflammatory Phenotypes and Biomarker Trajectories During Hospitalization for Acute Heart Failure

**DOI:** 10.3390/biomedicines14081795

**Published:** 2026-08-10

**Authors:** Raluca Ibănescu, Sebastian Ciurescu, Roxana Buzaș, Anda Gabriela Militaru, Marius Militaru, Diana-Alexandra Mîțu, Diana-Gabriela Ilaș, Elisabeta Trif, Marciana Ionela Boca, Daian-Ionel Popa, Daniel-Florin Lighezan

**Affiliations:** 1 Advanced Cardiology and Haemostaseology Research Centre, “Victor Babes” University of Medicine and Pharmacy, No. 2 Eftimie Murgu Square, 300041 Timisoara, Romania; raluca.ibanescu@umft.ro (R.I.); militaru.anda@umft.ro (A.G.M.); marius.militaru@umft.ro (M.M.); diana-alexandra.mitu@umft.ro (D.-A.M.); dlighezan@umft.ro (D.-F.L.); 2 Department V, Discipline of Medical Semiology I, “Victor Babes” University of Medicine and Pharmacy, No. 2 Eftimie Murgu Square, 300041 Timisoara, Romania; 3 Emergency Municipal Clinical Hospital, Gheorghe Dima Street Nr. 5, 300254 Timisoara, Romania; diana_ilas@yahoo.com (D.-G.I.); daian-ionel.popa@umft.ro (D.-I.P.); 4 Doctoral School, “Victor Babes” University of Medicine and Pharmacy, 300041 Timisoara, Romania; elisabeta.trif@umft.ro (E.T.); marciana.boca@umft.ro (M.I.B.); 5 Department of Neuroscience, University Clinic of Neurology II, “Victor Babes” University of Medicine and Pharmacy, E. Murgu Square, Nr. 2, 300041 Timisoara, Romania; 6 Institute for Cardiovascular Diseases of Timisoara, “Victor Babes” University of Medicine and Pharmacy, G.Adam Str. No. 13A, 300310 Timisoara, Romania; 7 Advanced Research Center of the Institute for Cardiovascular Diseases, “Victor Babes” University of Medicine and Pharmacy, No. 2 Eftimie Murgu Square, 300041 Timisoara, Romania; 8 Research Center for Medical Communication, “Victor Babes” University of Medicine and Pharmacy, 300041 Timisoara, Romania

**Keywords:** acute heart failure, C-reactive protein, procalcitonin, NT-proBNP, inflammatory phenotypes, biomarker trajectories, inflammation

## Abstract

**Background**: The clinical significance of inflammatory phenotypes and their temporal relationship with natriuretic peptide dynamics in acute heart failure (AHF) remain poorly defined. We investigated admission inflammatory phenotypes, in-hospital C-reactive protein (CRP) trajectories, and their relationship with N-terminal pro-B-type natriuretic peptide (NT-proBNP) dynamics. **Methods**: In this retrospective single-center cohort of 306 patients hospitalized for AHF, patients were stratified according to admission CRP (≤10 vs. >10 mg/L). Among patients with dual biomarker measurements (*n* = 147), a CRP–procalcitonin (PCT) concordance phenotype was evaluated. Serial CRP measurements were available in 65 patients, and NT-proBNP response (≥30% reduction) was assessed in 42 patients; because these repeat measurements were obtained at the treating physician’s discretion, the corresponding subgroups were older and more severely ill than the full cohort and are treated as exploratory. **Results**: Elevated admission CRP (>10 mg/L) was associated with higher NT-proBNP levels (3621 vs. 1251 pg/mL, *p* < 0.001), more severe symptoms (New York Heart Association [NYHA] III–IV: 45.9% vs. 29.8%, *p* = 0.006), and greater PCT positivity (21.8% vs. 8.7%, *p* = 0.029), independent of left-ventricular ejection fraction; the association persisted after adjustment for age, sex, and ejection fraction (adjusted odds ratio 2.40, 95% CI 1.27–4.55). The biomarker-defined CRP+/PCT+ phenotype showed the greatest concurrent NT-proBNP burden and the highest prevalence of heart failure with preserved ejection fraction (HFpEF, 70.6%). Despite declining NT-proBNP, CRP increased during hospitalization in 69.2% of patients (median ΔCRP +5.9 mg/L, *p* < 0.001), and the two changes were uncorrelated, indicating divergent biomarker trajectories. **Conclusions**: Admission CRP identifies patients with a more severe concurrent inflammatory and hemodynamic profile, while combined CRP–PCT phenotyping further characterizes this profile. Divergent CRP and NT-proBNP trajectories suggest that inflammatory and hemodynamic changes follow distinct time courses during AHF hospitalization. Because no post-discharge outcomes were available, these findings describe concurrent associations rather than prognosis.

## 1. Introduction

Acute heart failure (AHF) is one of the leading causes of emergency hospital admission among adults worldwide, accounting for more than one million hospital admissions annually in Europe. Despite contemporary management, in-hospital mortality remains at 4–7%, while 90-day rehospitalization exceeds 30% [1,2]. Contemporary concepts recognize AHF as a complex clinical syndrome in which hemodynamic, neurohormonal, metabolic, and inflammatory mechanisms interact throughout the course of acute decompensation [3]. Despite substantial advances in pharmacological and device-based treatment, this pathophysiological complexity continues to challenge risk stratification and therapeutic decision-making during the acute episode. Natriuretic peptides, particularly NT-proBNP, have become the cornerstone of AHF diagnosis and monitoring, but they primarily reflect ventricular wall stress and are insensitive to the inflammatory dimension of the decompensation syndrome [4]. In addition to natriuretic peptides, other cardiovascular biomarkers such as high-sensitivity cardiac troponin T (hs-cTnT) provide important prognostic information in heart failure, reflecting ongoing myocardial injury and identifying patients at increased risk of adverse outcomes, although they do not specifically characterize the inflammatory response [5].

Systemic inflammation has a complex, often overlooked role in AHF pathophysiology. Elevated C-reactive protein (CRP), an acute-phase reactant synthesized by the liver under interleukin-6 (IL-6) stimulation, is documented at hospital admission in a substantial proportion of AHF patients and is associated with worse in-hospital and post-discharge outcomes [6,7]. Multiple mechanisms may drive CRP elevation in this context: tissue hypoperfusion, gut ischemia with bacterial translocation, neurohumoral activation, and, in a clinically important subset, concurrent acute infection, which is itself a recognized precipitant of up to 30–40% of AHF hospitalizations [8,9]. Procalcitonin (PCT), a biomarker with high specificity for systemic bacterial infection, may help distinguish infectious from non-infectious triggers of AHF and, when interpreted together with CRP, may provide complementary inflammatory phenotyping [10,11]. However, the clinical profile and NT-proBNP burden of patients classified by a dual CRP + PCT phenotype have not been systematically characterized. Beyond admission values, little is known about how inflammatory biomarkers evolve during hospitalization or how these changes relate to established markers of hemodynamic recovery.

One particularly understudied aspect is the temporal behavior of CRP during AHF hospitalization. CRP peaks 24–72 h after the onset of the inflammatory trigger, introducing a systematic delay relative to the hemodynamic decompensation that precipitates admission. Whether CRP rises or falls during the hospitalization period and whether its trajectory is concordant with or dissociated from natriuretic peptide dynamics have not been systematically examined in large AHF cohorts. Understanding this kinetic dissociation is clinically relevant: a rising CRP in a hospitalized AHF patient might be misinterpreted as evidence of deterioration when it may simply reflect the natural temporal course of the acute-phase response initiated before admission.

Against this background, we sought to characterize inflammatory phenotypes in a contemporary cohort of patients hospitalized for AHF using admission CRP and a CRP–PCT concordance classification. We further examined serial CRP trajectories during hospitalization and explored their relationship with NT-proBNP dynamics. Finally, we assessed whether admission inflammatory phenotype was associated with in-hospital NT-proBNP reduction as an exploratory marker of hemodynamic response. We hypothesized that greater inflammatory activation at admission would identify patients with a more severe clinical profile, that combined CRP–PCT phenotyping would further distinguish infection-associated presentations, and that CRP and NT-proBNP would exhibit divergent trajectories during hospitalization.

## 2. Materials and Methods

### 2.1. Study Design and Setting

This retrospective, single-center, longitudinal observational cohort study was conducted at the Emergency Municipal Clinical Hospital, Timișoara, Romania. Consecutive adult patients admitted to the cardiology department with a primary diagnosis of acute heart failure (AHF) between January 2023 and December 2024 were evaluated for inclusion. This study was conducted and reported in accordance with the Strengthening the Reporting of Observational Studies in Epidemiology (STROBE) guidelines for cohort studies [12].

### 2.2. Study Population

**Figure 1:**
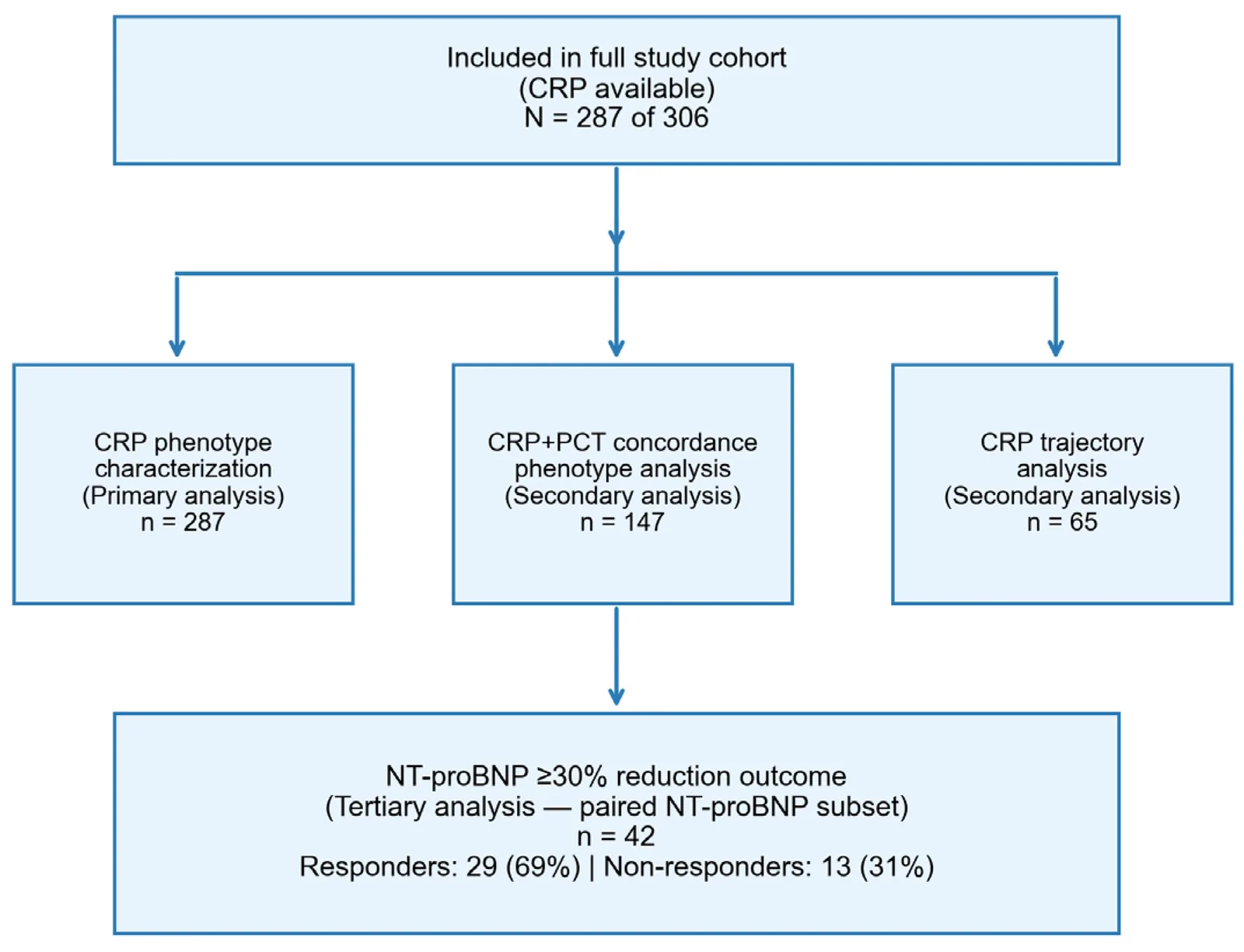
Patient selection flowchart and availability of biomarker data across the analysis subgroups (admission CRP, *n* = 287; paired CRP + PCT, *n* = 147; serial CRP, *n* = 65; serial NT-proBNP, *n* = 42).

Patients were eligible for inclusion if they: (1) were aged ≥18 years and (2) had a diagnosis of AHF meeting the 2021 ESC Heart Failure Guidelines criteria [13], requiring elevated natriuretic peptides together with objective clinical and/or radiographic congestion rather than natriuretic-peptide elevation alone. Admission CRP within 24 h and complete echocardiographic data were target variables sought for all patients rather than absolute inclusion filters; the number of patients contributing to each biomarker analysis therefore varies with data availability and is reported throughout (Figure 1). Patients were excluded if they had: (1) missing or incomplete core admission data precluding diagnosis; (2) an alternative primary diagnosis reclassified after initial admission (e.g., acute coronary syndrome or pulmonary embolism as the primary driver of the clinical presentation); or (3) duplicate records from prior admissions within the same hospitalization period. Heart failure phenotype was defined by admission left-ventricular ejection fraction (HFrEF < 40%, HFmrEF 40–49%, HFpEF ≥ 50%); systematic work-up for infiltrative or valvular etiologies of HFpEF (for example, amyloidosis screening or graded valvular assessment) was not part of routine care and could not be characterized.

After applying these criteria, 306 patients were included in the final cohort. Of these, 287 patients (93.8%) had a valid CRP measurement at admission and formed the primary analysis population for inflammatory phenotyping. A subset of 147 patients (48.0%) had both admission CRP and procalcitonin (PCT) measurements available and were eligible for the concordance phenotype analysis. Serial (admission and discharge) CRP measurements were available in 65 patients (21.2%) and serial NT-proBNP measurements suitable for response analysis were available in 42 patients (13.7%) (Figure 1). No formal a priori sample-size calculation was performed; all consecutive eligible patients admitted during the two-year study period were included, and the achievable sample size was therefore determined by the enrolment window.

### 2.3. Data Collection

Demographic, clinical, echocardiographic, and laboratory data were extracted from the institutional electronic medical records by trained data abstractors. Collected variables included: age, sex, body weight at admission and discharge, length of hospital stay, New York Heart Association (NYHA) functional class at admission and discharge, left-ventricular ejection fraction (LVEF) by transthoracic echocardiography, NT-proBNP at admission and discharge, serum CRP at admission and discharge (where available), serum PCT at admission (where available), admission leukocyte count, serum uric acid at admission, and pharmacological treatment. Pharmacological data comprised pre-admission (chronic) use of furosemide and guideline-directed medical therapy for heart failure, including angiotensin-converting-enzyme inhibitors or angiotensin-receptor blockers, angiotensin-receptor–neprilysin inhibitors, beta-blockers, mineralocorticoid-receptor antagonists, and sodium-glucose cotransporter-2 inhibitors. Comorbidities recorded included hypertension, diabetes mellitus, dyslipidemia, active smoking, and alcohol use.

Heart failure phenotype was classified according to current ESC criteria based on the admission LVEF: HF with reduced ejection fraction (HFrEF, LVEF < 40%), HF with mildly reduced ejection fraction (HFmrEF, LVEF 40–49%), and HF with preserved ejection fraction (HFpEF, LVEF ≥ 50%) [13].

### 2.4. Inflammatory Biomarker Assessment and Phenotyping

Serum CRP was measured by a high-sensitivity immunoturbidimetric assay. Serum PCT was measured by an electrochemiluminescence immunoassay (ECLIA). All laboratory determinations were performed in the hospital’s accredited central laboratory using standardized protocols.

CRP phenotype (primary inflammatory classification): Patients were stratified into two groups based on admission CRP: CRP-low (CRP ≤ 10 mg/L) and CRP-high (CRP > 10 mg/L). The threshold of 10 mg/L was selected as a clinically established cutoff for systemic inflammation and has been widely used in acute cardiovascular settings [6,14].

CRP + PCT concordance phenotype (secondary inflammatory classification): Among patients with both admission CRP and PCT available (*n* = 147), four mutually exclusive biomarker-defined phenotypes were defined by crossing CRP positivity (CRP > 10 mg/L) and PCT positivity (PCT ≥ 0.5 ng/mL, the standard threshold for bacteremia risk) [10]: Group A (CRP−/PCT−); Group B (CRP−/PCT+); Group C (CRP+/PCT−); and Group D (CRP+/PCT+). These are biomarker-defined categories only. Because confirmed infection diagnoses, microbiological results, imaging, antibiotic exposure, and renal function were not available in this dataset, the groups are not equated with confirmed clinical infection; the admission leukocyte count is reported alongside them as the single available infection-relevant variable.

As this was a retrospective study, biomarker testing was performed at the discretion of the treating physician according to routine clinical practice rather than a predefined study protocol. Consequently, CRP, PCT, and NT-proBNP measurements were not available for all patients, and analyses involving these biomarkers were performed using the corresponding available-data subsets. Because repeat and PCT testing was discretionary, patients with these measurements were compared with those without to assess selection (Supplementary Table S1).

In-hospital CRP trajectory: In patients with both admission and discharge CRP measurements available (*n* = 65), the within-patient change in CRP (ΔCRP = CRP_discharge − CRP_admission) was calculated. Patients were classified as showing a rising or falling CRP trajectory based on the sign of ΔCRP. The Wilcoxon signed-rank test was used to assess whether the median ΔCRP differed significantly from zero.

### 2.5. Outcome Measures

Among the 306 enrolled patients, 42 had paired NT-proBNP measurements at both admission and discharge and constituted the analysis population for this outcome. By construction, the serial CRP and paired NT-proBNP analyses included only patients who survived to a second in-hospital measurement; patients who died in hospital could not contribute paired data.

The primary analytical objectives were: (1) characterization of the CRP distribution at admission across HF phenotypes (HFrEF, HFmrEF, HFpEF) and NYHA functional classes; (2) comparison of clinical, hemodynamic, and laboratory characteristics across CRP inflammatory phenotype groups (*n* = 287); (3) comparative profile of the four CRP + PCT concordance phenotypes (*n* = 147); and (4) the in-hospital CRP trajectory (rising vs. falling) in the paired-measurement subset (*n* = 65). NT-proBNP ≥30% reduction was examined as an exploratory endpoint (*n* = 42); given the limited sample size, a post hoc power estimate indicates that this subset could detect an OR of approximately 4.0 or greater at 80% power (two-sided α = 0.05, 69% event rate), and therefore moderate-sized associations cannot be excluded.

### 2.6. Statistical Analysis

Continuous variables are reported as mean ± standard deviation (SD) when approximately normally distributed or as median with interquartile range [IQR] for non-normally distributed variables. The Shapiro–Wilk test was used to assess normality. Categorical variables are reported as counts and percentages.

Comparisons between two independent groups were performed using the Mann–Whitney U test (with normal approximation) for continuous non-normally distributed variables, the two-sample *t*-test for normally distributed variables, and Pearson’s chi-square test or Fisher’s exact test (when expected cell counts were <5) for categorical variables. In patients with serial measurements, within-patient changes from admission to discharge were assessed using the Wilcoxon signed-rank test. The same tests were used for the pre-specified subgroup comparisons (HFpEF versus combined HFrEF/HFmrEF, and across the four CRP + PCT phenotype groups); no formal interaction terms were fitted given the sample size.

For the NT-proBNP ≥30% reduction outcome, binary logistic regression was used to estimate unadjusted odds ratios (ORs) with 95% confidence intervals (CIs). Given the limited sample size of the paired subset (*n* = 42) and the exploratory nature of this analysis, only univariable associations were examined for this endpoint. As a sensitivity analysis for the principal association, a multivariable logistic regression was fitted in the larger CRP subset for above-median admission NT-proBNP, with CRP-high, age, sex, and LVEF as covariates. The covariates (age, sex, and LVEF) were selected a priori as recognized clinical determinants of natriuretic-peptide concentration rather than by stepwise selection.

All tests were two-sided, and a *p*-value < 0.05 was considered statistically significant. Owing to the exploratory and hypothesis-generating nature of subgroup analyses, no correction for multiple comparisons was applied; results of secondary analyses should be interpreted accordingly. Statistical analyses were performed using Python 3 (version 3.11) with custom implementations of standard nonparametric tests verified against published formulas [15,16].

Missing data were not imputed. Analyses were restricted to patients with complete data for the variable of interest, and the number of patients contributing to each analysis is reported throughout.

### 2.7. Ethics

This study was approved by the Research Ethics Committee of the “Victor Babeș” University of Medicine and Pharmacy, Timișoara, Romania (approval No. 02, dated 8 January 2026, revised 14 July 2026; affiliated hospital approval E 3203/2026), which expressly covered the retrospective use of anonymized records collected between January 2023 and December 2024. Given the retrospective nature of the study and the use of anonymized data from the institutional electronic records, the requirement for individual informed consent was waived. The study was conducted in accordance with the principles of the Declaration of Helsinki.

## 3. Results

### 3.1. Baseline Characteristics of the Study Pupolation

A total of 306 patients hospitalized for AHF between January 2023 and December 2024 met the inclusion criteria and were included in the study (Figure 1). The mean age was 70.7 ± 10.9 years, and 158 patients (51.6%) were male. The mean length of hospital stay was 8.2 ± 3.6 days. At admission, 7 patients (2.3%) were classified as NYHA class I, 189 (61.8%) as class II, 99 (32.4%) as class III, and 11 (3.6%) as class IV; the NYHA I patients all met the 2021 ESC criteria on the basis of elevated natriuretic peptides with objective congestion, their low functional class reflecting assessment after initial decongestion rather than absence of decompensation. The mean LVEF was 49.7 ± 12.6%; 61 patients (20.0%) had HFrEF, 75 (24.6%) had HFmrEF, and 169 (55.4%) had HFpEF. Among patients with available measurements, the median admission NT-proBNP concentration was 1778.5 pg/mL (IQR 457.0–5838.0; *n* = 238), while the median CRP concentration was 7.2 mg/L (IQR 2.2–16.1; *n* = 287). Hypertension was present in 274 patients (89.5%), dyslipidemia in 259 (84.6%), diabetes mellitus in 115 (37.6%), active smoking in 94 (30.7%), and alcohol use in 95 (31.0%). Pre-admission furosemide was recorded in 238 patients (78.3%); this reflects chronic outpatient use, and essentially all patients received intravenous diuretic therapy during the admission. Baseline characteristics, including guideline-directed medical therapy, are summarized in Table 1.

### 3.2. CRP Inflammatory Phenotype: Distribution and Clinical Correlates

**Figure 2:**
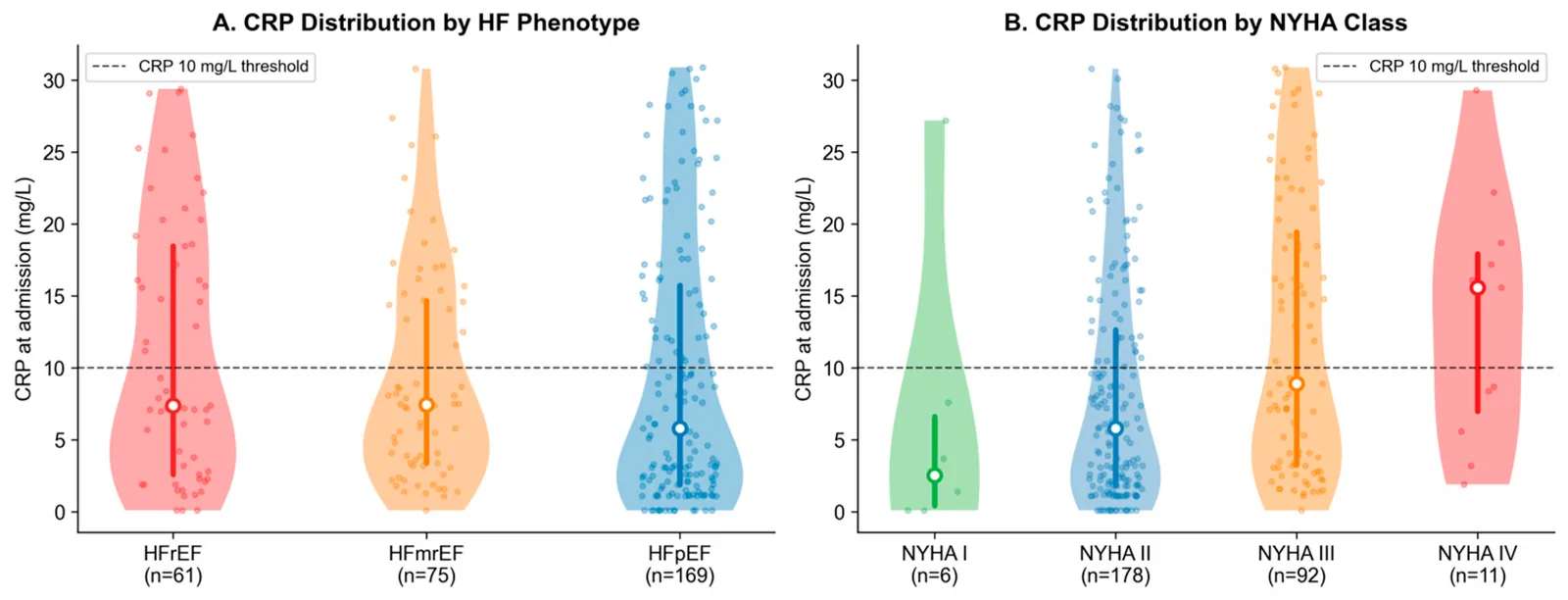
Distribution of admission C-reactive protein (CRP) according to (**A**) heart failure phenotype (HFrEF, HFmrEF, HFpEF) and (**B**) NYHA functional class. The CRP axis is not truncated; extreme values are displayed, and outliers beyond the whiskers are plotted individually.

Among the 287 patients with available admission CRP measurements, 178 (62.0%) were classified as CRP-low (≤10 mg/L) and 109 (38.0%) as CRP-high (>10 mg/L). Admission CRP did not differ significantly across heart failure phenotypes (Figure 2A), although values tended to increase with worsening NYHA functional class (Figure 2B).

Patients in the CRP-high group exhibited a more severe clinical profile at admission than those in the CRP-low group (Table 2). Despite these differences, left-ventricular ejection fraction and heart failure phenotype distribution were similar between groups. Median NT-proBNP levels were significantly higher in the CRP-high group (3621.5 pg/mL [IQR 997.0–8179.0]) than in the CRP-low group (1251.0 pg/mL [IQR 290.0–4599.0]; *p* < 0.001). NYHA class III–IV was more prevalent in the CRP-high group (45.9% vs. 29.8%, *p* = 0.006). PCT positivity (≥0.5 ng/mL) was significantly more frequent in the CRP-high group (21.8% vs. 8.7%, *p* = 0.029). Pre-admission furosemide use was higher in the CRP-high group (86.2% vs. 72.2%, *p* = 0.006). Conversely, hypertension was less prevalent in the CRP-high group (85.3% vs. 93.3%, *p* = 0.028). No significant differences were observed in age, LVEF, heart failure phenotype distribution, diabetes mellitus, or dyslipidemia between the two groups (all *p* > 0.05). When HFrEF and HFmrEF were combined and compared with HFpEF, admission CRP and the proportion with CRP >10 mg/L did not differ (5.8 vs. 7.4 mg/L, *p* = 0.15; 37.7% vs. 38.6%, *p* = 0.88), whereas NT-proBNP and NYHA III–IV were lower in HFpEF (*p* < 0.001), indicating that the inflammatory signal is independent of the preserved versus reduced ejection fraction distinction. In a multivariable logistic model (*n* = 225), CRP >10 mg/L remained independently associated with above-median admission NT-proBNP after adjustment for age, sex, and LVEF (adjusted OR 2.40, 95% CI 1.27–4.55, *p* = 0.007).

### 3.3. CRP + PCT Concordance Phenotype

Among the 147 patients with both admission CRP and PCT measurements available, four inflammatory concordance phenotypes were identified: Group A (CRP−/PCT−, *n* = 63, 42.9%), Group B (CRP−/PCT+, *n* = 6, 4.1%), Group C (CRP+/PCT−, *n* = 61, 41.5%), and Group D (CRP+/PCT+, *n* = 17, 11.6%) (Table 3).

**Figure 3:**
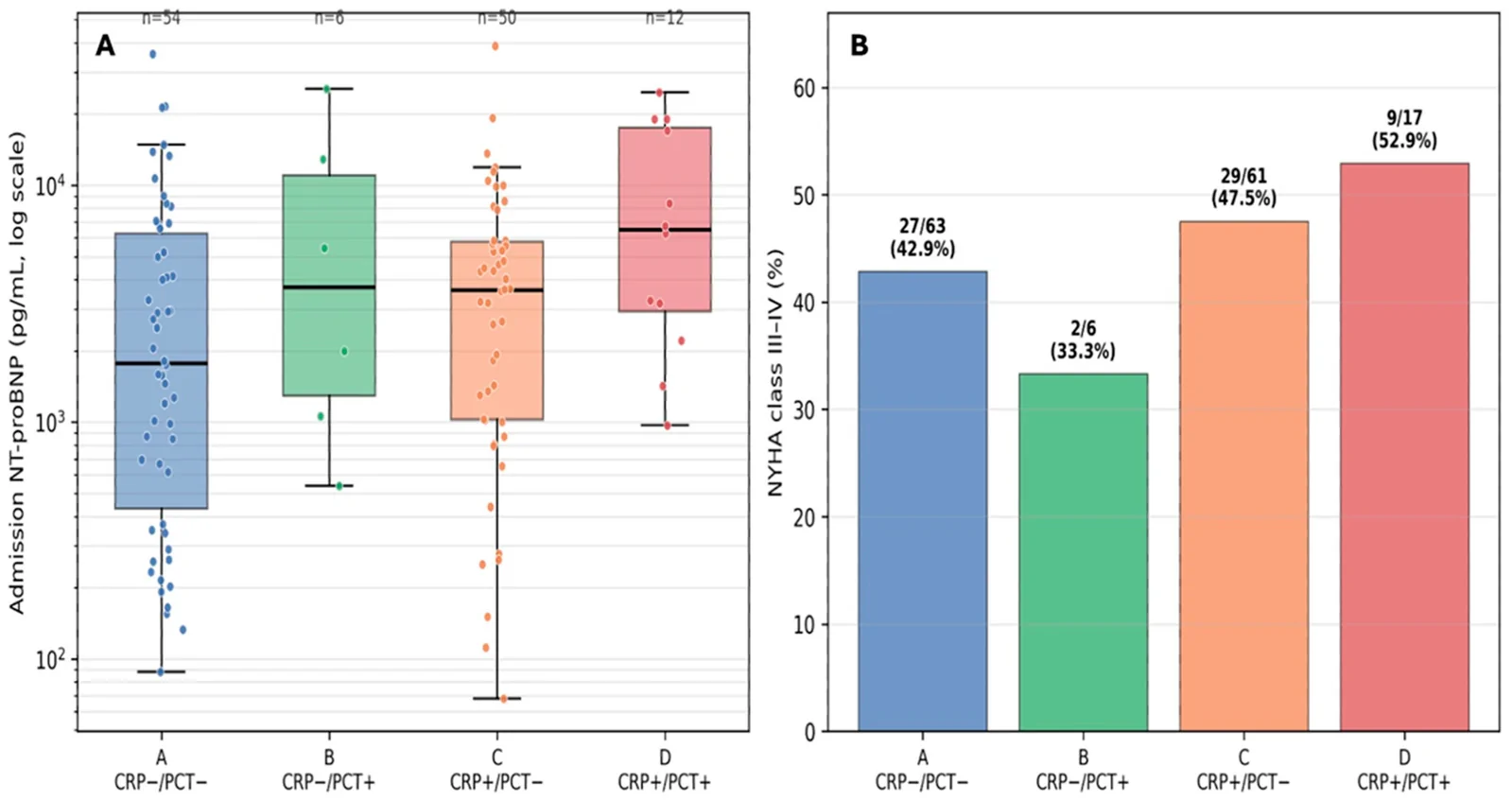
(**A**) Admission NT-proBNP distribution (box-and-whisker, log scale) and (**B**) prevalence of NYHA class III–IV across the four biomarker-defined CRP + PCT concordance phenotypes. Percentages in panel B are shown with numerators and denominators; several groups are small (Group B *n* = 6, Group D *n* = 17), so proportions should be interpreted cautiously.

Admission NT-proBNP levels increased progressively across the four biomarker-defined phenotypes, with the lowest values observed in Group A and the highest in Group D (Figure 3A). Groups B and C exhibited intermediate NT-proBNP levels. Group D also had the highest prevalence of HFpEF (70.6%). Admission leukocyte counts rose in parallel across the phenotypes (median 8.5 × 10^9^/L in Group A, 10.6 in Group C, and 15.6 in Group D; Table 3) and were higher in CRP-high than CRP-low patients overall (10.2 vs. 7.9 × 10^9^/L, *p* < 0.001), consistent with, but not diagnostic of, a greater inflammatory or infectious burden in the dual-positive group. The proportion of patients with NYHA class III–IV was similar across all four phenotypes (Figure 3B).

### 3.4. In-Hospital CRP and NT-proBNP Trajectories

**Figure 4:**
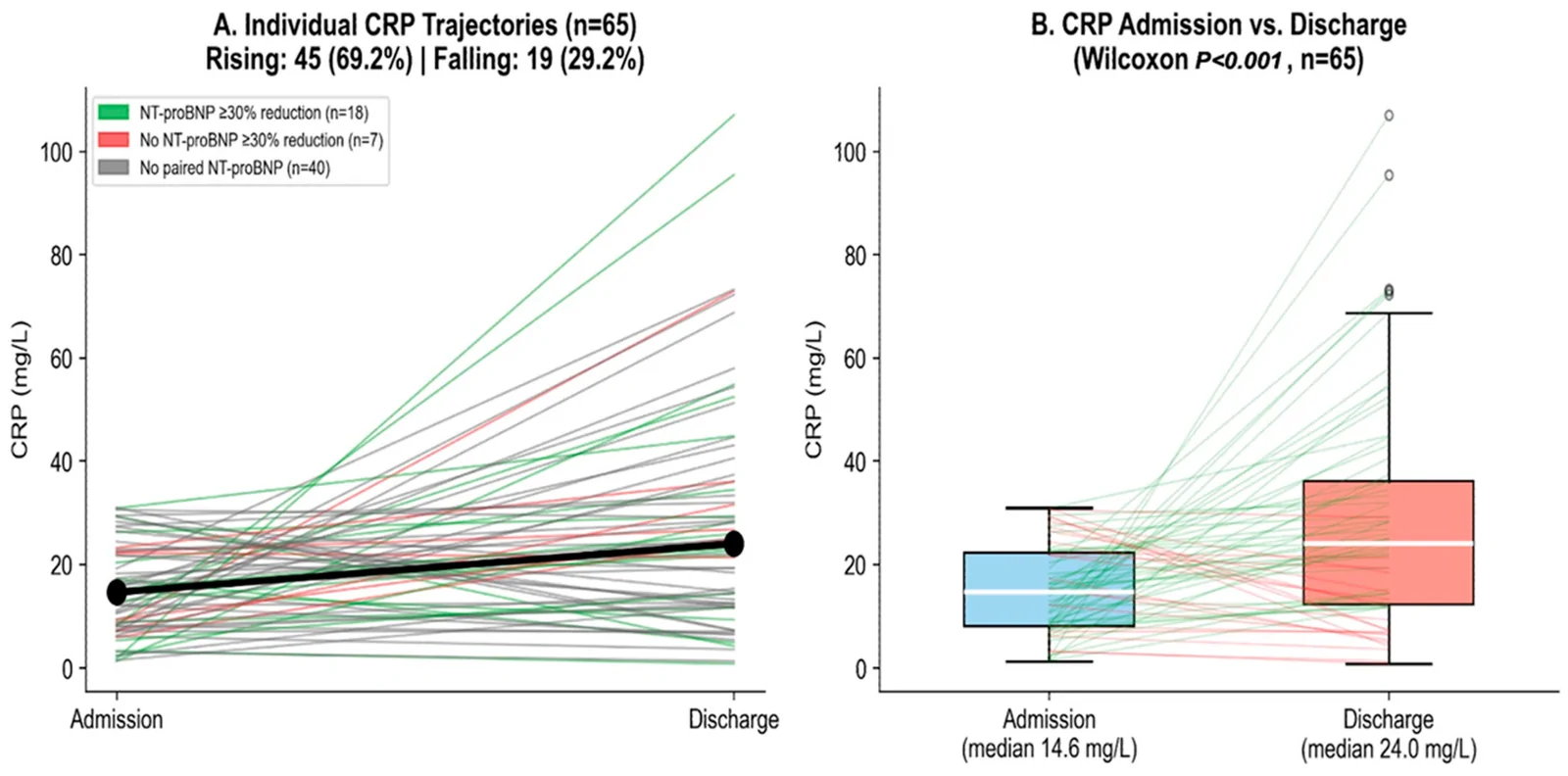
In-hospital C-reactive protein (CRP) trajectory from admission to discharge in patients with paired measurements (*n* = 65). Individual patient trajectories are shown as thin lines, with the black markers representing the median CRP values at admission and discharge.

Serial CRP measurements at both admission and discharge were available for 65 patients (21.2% of the cohort). These patients were older and had markedly higher admission CRP, NT-proBNP, and NYHA class than those without serial measurements (Supplementary Table S1), so the trajectory findings describe a selected, higher-acuity subgroup and are exploratory. Overall, CRP increased significantly during hospitalization, from a median of 14.6 mg/L (IQR 8.1–22.2) at admission to 24.0 mg/L (IQR 12.3–36.0) at discharge (Wilcoxon’s signed-rank test, *p* < 0.001), corresponding to a median ΔCRP of +5.9 mg/L (IQR −1.5 to +26.0). At the individual level, 45 patients (69.2%) exhibited a rising CRP trajectory, 19 (29.2%) a declining trajectory, and 1 (1.5%) no change (Figure 4). The two measurements were obtained a mean of 8.2 ± 3.6 days apart, without recorded exact intervals. Among the 26 patients with paired CRP and NT-proBNP measurements (complete numeric values in 25), CRP rose (median ΔCRP +10.7 mg/L) while NT-proBNP fell (median ΔNT-proBNP −2036 pg/mL), the two changes were uncorrelated (Spearman ρ = 0.11, *p* = 0.61), and CRP increased despite a concurrent NT-proBNP reduction in 14 of 25 patients, illustrating divergent in-hospital biomarker trajectories.

### 3.5. NT-proBNP ≥ 30% Reduction: Exploratory Clinical Improvement Endpoint

Among the 42 patients with paired NT-proBNP measurements at both admission and discharge, 29 (69.0%) achieved an NT-proBNP ≥ 30% reduction and 13 (31.0%) did not. Baseline characteristics of responders and non-responders are presented in Table 4.

The only variable that differed significantly between groups was NT-proBNP at admission: patients who achieved a ≥30% reduction had significantly higher admission NT-proBNP [median 6586.0 pg/mL, IQR 3176.0–14,616.0] compared with non-responders [median 3293.0 pg/mL, IQR 809.0–5838.0] (Mann–Whitney‘s U test, *p* = 0.031). This finding is consistent with a regression-to-mean phenomenon, in which patients presenting with more extreme baseline values are more likely to cross the ≥30% reduction threshold regardless of treatment effect.

**Figure 5:**
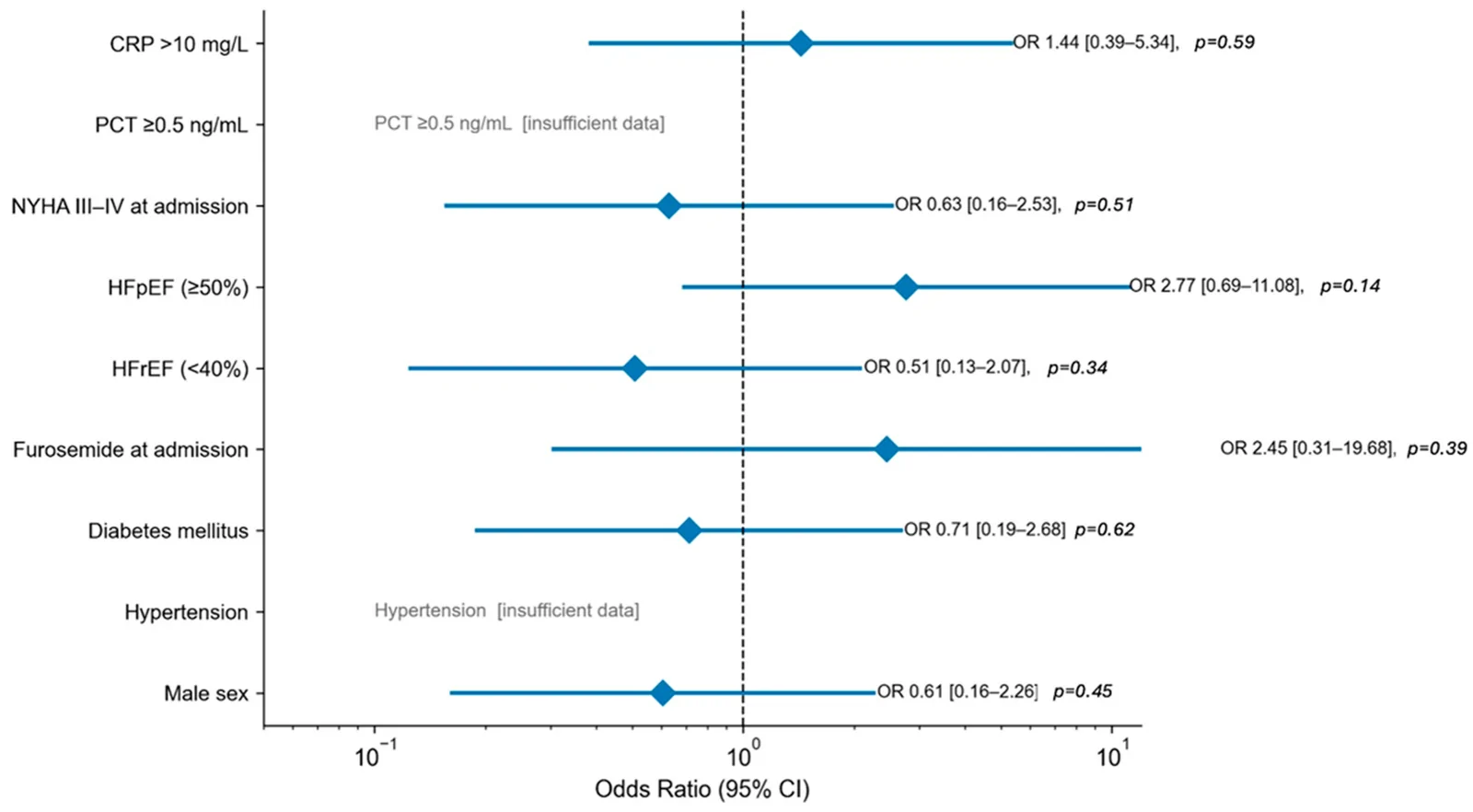
Univariable associations between baseline characteristics and NT-proBNP ≥ 30% reduction, expressed as odds ratios with 95% confidence intervals (*n* = 42).

Similarly, admission PCT, PCT positivity, LVEF, HF phenotype, NYHA class, sex, body weight, pre-admission furosemide use, and comorbidities were not significantly associated with NT-proBNP response (all *p* > 0.05; Table 4, Figure 5). No class of guideline-directed medical therapy differed between responders and non-responders (all *p* > 0.6). These results should be interpreted in the context of the limited sample size (*n* = 42), which precluded multivariable analysis and may have been insufficient to detect moderate-sized effects.

## 4. Discussion

This study investigated the inflammatory landscape of AHF hospitalization through three complementary lenses: the clinical correlates of the CRP phenotype at admission, the profile of a four-group CRP + PCT concordance classification, and the in-hospital dynamics of CRP relative to NT-proBNP-based hemodynamic change. Four main findings emerged. CRP > 10 mg/L, present in 38% of patients, identified a more severe clinical profile at admission: higher NT-proBNP, more frequent NYHA class III–IV, and greater PCT positivity, with no difference in LVEF or HF structural phenotype, and this association persisted after multivariable adjustment. In the dual-biomarker subset, the CRP+/PCT+ concordance phenotype (Group D) had the greatest concurrent NT-proBNP burden and the highest prevalence of HFpEF. CRP rose during hospitalization in 69.2% of patients despite concurrent NT-proBNP reduction, revealing a kinetic dissociation between the two biomarkers. Finally, in the exploratory subset with paired NT-proBNP measurements (*n* = 42), admission NT-proBNP was the only variable associated with a ≥30% reduction during hospitalization, most likely reflecting regression to the mean. Because no post-discharge outcomes were available, these are associations with concurrent severity, not measures of prognosis or risk.

### 4.1. CRP as a Severity Surrogate in AHF: Comparison with the Published Literature

Alonso-Martínez et al. [6] demonstrated that CRP at hospital admission independently predicted short-term mortality and re-hospitalization in patients with decompensated HF, establishing a foundational link between systemic inflammation and adverse outcomes in this population. Our findings support the severity–CRP relationship from a different angle: in a contemporary cohort managed under current ESC guidelines, CRP > 10 mg/L identified a clinically distinct subgroup, one with nearly threefold higher NT-proBNP and a greater prevalence of severe functional impairment (NYHA III–IV) compared with the CRP-low group. This association was independent of the preserved versus reduced ejection fraction distinction: combining HFrEF and HFmrEF and comparing them with HFpEF revealed no difference in admission CRP, indicating that the inflammatory signal tracks decompensation severity rather than structural phenotype.

The correlation between CRP and NT-proBNP across phenotypic groups supports the inflammatory–neurohormonal interplay that characterizes AHF decompensation [5]. Clinically, CRP and PCT are inexpensive and routinely available and may help characterize the inflammatory profile accompanying an acute heart failure admission; whether they predict post-discharge outcomes was not testable in this cohort and requires prospective study with follow-up. We therefore avoid the term risk stratification, which our data cannot support.

The high prevalence of hypertension observed in our cohort is consistent with its role as a leading substrate for heart failure in this population, in which arterial hypertension frequently coexists with other cardiovascular risk factors, including elevated serum uric acid, particularly among elderly patients. The higher prevalence of PCT positivity (≥0.5 ng/mL) in the CRP-high group (21.8% vs. 8.7%, *p* = 0.029) suggests that a subset of CRP-high patients had a concurrent infectious or bacterial contribution to their inflammatory burden, although this was not confirmed clinically. Infection is a well-recognized precipitant of AHF decompensation, and the combined CRP + PCT approach helps characterize this dimension beyond either marker alone [8].

### 4.2. CRP + PCT Concordance Phenotype: A Multi-Dimensional Inflammatory Classification

The four-group CRP + PCT concordance phenotype represents a step beyond single-biomarker stratification. Its gradient, with NT-proBNP increasing from Group A (neither marker elevated) through Group C (CRP elevated) to Group D (both elevated), maps onto a biologically coherent spectrum of inflammatory activation in AHF mirrored by a parallel rise in admission leukocyte count.

Several observations from Group D (CRP+/PCT+, *n* = 17) stand out. Despite representing only 11.6% of patients with dual biomarker data, this group had a median NT-proBNP nearly four times higher than Group A (6511.5 vs. 1778.5 pg/mL), the highest prevalence of HFpEF (70.6%), and the highest admission leukocyte count. Because this phenotype is defined by biomarker thresholds alone, it should not be equated with confirmed infection, which was not ascertainable in this dataset; the elevated leukocyte count is supportive but not diagnostic. The overrepresentation of HFpEF here is biologically plausible, as patients with HFpEF often exhibit impaired diastolic reserve and a higher burden of metabolic comorbidity, rendering them more vulnerable to decompensation during systemic inflammation or infection [17]. Importantly, because bacterial infection and sepsis can raise NT-proBNP independently of ventricular wall stress, the higher NT-proBNP in the CRP+/PCT+ phenotype may partly reflect infection-associated elevation rather than heart failure severity alone. The CRP + PCT combination may therefore identify an AHF subset in which an infectious contribution warrants clinical evaluation, although this requires prospective confirmation with microbiological and clinical adjudication.

By contrast, Group B (CRP−/PCT+, isolated PCT elevation, *n* = 6) was too small in our cohort to draw meaningful conclusions. The rarity of this phenotype, low-grade bacteremia in the absence of a strong CRP response, may reflect early infection prior to full acute-phase response maturation, host immunosuppression, or PCT elevation driven by non-infectious causes (cardiac shock, corticosteroid use) [10]. The concordance phenotype framework has methodological limitations in this regard: Group B is intrinsically likely to be small in any AHF cohort where bacterial co-infection typically triggers both PCT and CRP, and future studies with larger samples will be needed to characterize this group reliably.

### 4.3. In-Hospital CRP Kinetics: A Peak-Lag Interpretation

The fact that CRP rose in 69.2% of patients despite concurrent NT-proBNP reduction is a clinically relevant observation, indicating divergent in-hospital trajectories of inflammatory and hemodynamic biomarkers. The divergence is consistent with the distinct biological kinetics of the two biomarkers. NT-proBNP responds rapidly to changes in ventricular wall stress and hemodynamic unloading, whereas CRP reflects the hepatic acute-phase response and typically peaks 24–72 h after the inflammatory trigger [14]. In AHF, the decompensation and its inflammatory trigger usually precede hospitalization by hours to days, so patients may be admitted while NT-proBNP is already elevated but CRP is still rising. However, because only two non-standardized measurements were available per patient, because a mean of 8.2 ± 3.6 days apart was obtained without recorded exact intervals, and because the in-subset change in CRP was uncorrelated with the change in NT-proBNP (Spearman’s ρ = 0.11, *p* = 0.61), these data cannot establish a defined CRP peak-lag sequence. We therefore present the divergence as a hypothesis-generating observation of biomarker dissociation rather than as an expected clinical pattern. A practical corollary, which also requires prospective testing, is that a rising CRP during early AHF hospitalization should not automatically be read as deterioration and that serial CRP may be most informative if sampled later in the admission.

### 4.4. NT-proBNP ≥ 30% Reduction: Limitations and Contextual Interpretation

This pattern reflects regression to the mean: patients with very high baseline values have a greater arithmetic chance of crossing a proportional threshold, regardless of the underlying treatment effect [18]. The clinical meaning of an in-hospital NT-proBNP fall is itself uncertain: NT-proBNP-guided therapy has not consistently improved outcomes in randomized trials, and a reduction does not reliably indicate a better prognosis, nor does its absence reliably indicate a worse one [19,20]. We therefore treat the ≥30% reduction strictly as an exploratory biochemical endpoint, not a validated surrogate of clinical improvement.

The absence of an association between admission CRP and NT-proBNP response should therefore not be interpreted as evidence that inflammation is unrelated to AHF outcomes. Rather, it likely reflects the distinct biological processes captured by these biomarkers and their divergent in-hospital trajectories, as demonstrated in the CRP trajectory analysis. A properly powered study, with serial measurements obtained at multiple time points and a composite outcome combining hemodynamic, functional, and inflammatory response, would be needed to determine whether the inflammatory phenotype at admission predicts subsequent clinical improvement.

### 4.5. Limitations

First, the retrospective single-center design introduces potential selection bias and limits generalizability to other healthcare settings or geographic populations. The cohort was nonetheless large for a single Romanian academic center, with consecutive enrolment over two years under standardized ESC guidelines-based management.

Second, the availability of serial biomarker measurements was limited and discretionary: CRP pairs were available in 65 patients (21.2%) and NT-proBNP pairs in 42 (13.7%), with no standardised protocol or timing for repeat or PCT testing. Patients selected for these measurements were significantly older and had higher CRP, NT-proBNP, and NYHA class than those without (Supplementary Table S1), so the trajectory and response analyses reflect a higher-acuity, selected subset. In addition, these serial analyses necessarily included only patients who survived to a second measurement, which may under-represent the sickest patients and biases the findings toward survivors.

Third, the four concordance groups are defined by biomarker thresholds alone. Confirmed infection diagnoses, microbiology, fever, imaging, and antibiotic exposure were not available, so Group D should not be read as confirmed infection; the admission leukocyte count is the only supporting infection-relevant variable. PCT ordering was itself driven by clinical suspicion, which likely enriched the tested subgroup for higher acuity.

Fourth, the study lacked post-discharge follow-up data. Mortality, readmission, and functional outcomes could not be assessed, so no prognostic or risk-stratification claims can be made; all associations reported are with concurrent severity.

Fifth, several variables that influence the biomarkers studied were not recorded. Heart-failure etiology, ventricular arrhythmia burden, renal function, hemoglobin/anemia, and confirmed infection status were unavailable; the absence of renal function is particularly relevant because impaired clearance can elevate both NT-proBNP and PCT and could not be adjusted for. Prior heart failure hospitalizations were also not captured, so we could not examine whether recurrent admissions, which carry a worse prognosis and may reflect persistent inflammatory activation [21], contributed to the inflammatory profile observed.

Sixth, a conceptual limitation concerns the diagnosis itself. Natriuretic peptides are sensitive but not specific, and pneumonia, sepsis, renal dysfunction, or atrial fibrillation can raise NT-proBNP without primary cardiac decompensation. Because our cohort was identified as AHF partly on the basis of natriuretic peptide elevation, part of the association between inflammatory markers and higher NT-proBNP may reflect the severity of systemic illness rather than a heart-failure-specific inflammatory phenotype. This concern is greatest for HFpEF, which remains a diagnostically heterogeneous construct in which objective confirmation of elevated filling pressures and exclusion of non-cardiac causes are frequently incomplete [22,23]; some patients labelled HFpEF may have had a primary non-cardiac acute illness with secondary natriuretic-peptide elevation, and the HFpEF-related findings should be read with this caveat.

Finally, given the marked heterogeneity of AHF across ejection-fraction phenotypes and the incomplete availability of several variables, the study was likely underpowered to detect modest between-group differences, and non-significant comparisons should not be read as evidence of absence. The small paired subsets could not support multivariable modelling; the single multivariable sensitivity analysis was confined to the larger CRP subset, and residual confounding cannot be excluded. In-hospital weight change was recorded in a coarsely rounded range and should be read as an approximate decongestion signal rather than a precise fluid loss measurement.

## 5. Conclusions

In this retrospective cohort of 306 patients hospitalized for acute heart failure (AHF), elevated admission CRP (>10 mg/L) was associated with a more severe concurrent clinical profile, characterized by higher NT-proBNP levels, greater functional impairment, and more frequent PCT positivity, independent of left-ventricular ejection fraction and robust to multivariable adjustment. The biomarker-defined CRP + PCT concordance phenotype further characterized this profile, with the dual-positive Group D showing the greatest concurrent natriuretic peptide burden, the highest prevalence of HFpEF, and the highest leukocyte count. During hospitalization, CRP and NT-proBNP followed discordant, uncorrelated trajectories, with CRP increasing in most patients despite a reduction in NT-proBNP. These findings suggest that admission CRP may add complementary information about the inflammatory profile accompanying AHF and that CRP and natriuretic peptides reflect distinct biological processes during acute decompensation. Whether these phenotypes predict post-discharge outcomes was not evaluable in this cohort and requires prospective study.

## Figures and Tables

**Table 1:** Baseline demographic, clinical, echocardiographic, and laboratory characteristics of the overall study cohort (*n* = 306).

Variable	Value (*n* = 306)
Demographics	
Age (years), mean ± SD	70.7 ± 10.9
Male sex, *n* (%)	158 (51.6%)
Clinical presentation	
Admission NYHA NYHA I, *n* (%)	7 (2.3%)
Admission NYHA NYHA II, *n* (%)	189 (61.8%)
Admission NYHA NYHA III, *n* (%)	99 (32.4%)
Admission NYHA NYHA IV, *n* (%)	11 (3.6%)
Body weight at admission (kg), mean ± SD	84.6 ± 16.3
In-hospital weight change (kg), mean ± SD	−3.5 ± 1.1
Cardiac phenotype	
LVEF at admission (%), mean ± SD	49.7 ± 12.6
HFrEF (LVEF < 40%), *n* (%)	61 (20.0%)
HFmrEF, *n* (%)	75 (24.6%)
HFpEF, *n* (%)	169 (55.4%)
Biomarkers	
NT-proBNP at admission (pg/mL), median [IQR]	1778.5 [457, 5838.0] (*n* = 238)
CRP at admission (mg/L), median [IQR]	7.2 [2.2, 16.1] (*n* = 287)
CRP > 10 mg/L, *n* (%)	109 (38.0%)
Procalcitonin at admission (ng/mL), median [IQR]	0.0 [0.0, 0.2] (*n* = 149)
PCT ≥ 0.5 ng/mL, *n* (%) of those tested	23/149 (15.4%)
Comorbidities	
Hypertension, *n* (%)	274 (89.5%)
Diabetes mellitus, *n* (%)	115 (37.6%)
Dyslipidemia, *n* (%)	259 (84.6%)
Active smoking, *n* (%)	94 (30.7%)
Alcohol use, *n* (%)	95 (31.0%)
Treatment	
Pre-admission furosemide, *n* (%)	238 (78.3%)
Length of stay (days), mean ± SD	8.2 ± 3.6
Guideline-directed therapy	
Beta-blocker, *n* (%)	271 (88.6%)
MRA (spironolactone), *n* (%)	205 (67.0%)
ACE inhibitor/ARB, *n* (%)	187 (61.1%)
SGLT2 inhibitor, *n* (%)	78 (25.5%)
ARNI (sacubitril/valsartan), *n* (%)	30 (9.8%)

Note: Values are mean ± SD, median [IQR], or *n* (%); the number of contributing observations is shown where data were incomplete. Pre-admission furosemide denotes chronic outpatient use; nearly all patients received intravenous diuretics in hospital. In-hospital weight change was recorded in a coarsely rounded range and approximates decongestion. Abbreviations: NYHA, New York Heart Association; LVEF, left-ventricular ejection fraction; HFrEF/HFmrEF/HFpEF, heart failure with reduced/mildly reduced/preserved ejection fraction; NT-proBNP, N-terminal pro-B-type natriuretic peptide; CRP, C-reactive protein; PCT, procalcitonin; MRA, mineralocorticoid-receptor antagonist; ARB, angiotensin-receptor blocker; ARNI, angiotensin-receptor–neprilysin inhibitor; SGLT2, sodium-glucose cotransporter-2.

**Table 2:** Clinical, hemodynamic, and laboratory characteristics by CRP inflammatory phenotype (CRP-low, ≤10 mg/L vs. CRP-high, >10 mg/L; *n* = 287).

Variable	Low CRP < 10 mg/L (*n* = 178)	High CRP > 10 mg/L (*n* = 109)	*p*-Value
Age (yrs), mean ± SD	70.2 ± 10.3	71.6 ± 11.8	0.083
HFrEF, *n* (%)	32 (18.1%)	25 (22.9%)	0.318
HFmrEF, *n* (%)	46 (26.0%)	24 (22.0%)	0.448
HFpEF, *n* (%)	99 (55.9%)	60 (55.0%)	0.884
LVEF (%), mean ± SD	49.6 ± 12.1	49.7 ± 13.7	0.569
NYHA III–IV, *n* (%)	53 (29.8%)	50 (45.9%)	**0.006**
NT-proBNP (pg/mL), median [IQR]	1251.0 [290, 4599.0] (*n* = 142)	3621.5 [997, 8179.0] (*n* = 84)	**<0.001**
PCT > 0.5 ng/mL, *n* (%)	6 (8.7%)	17 (21.8%)	**0.029**
Pre-admission furosemide, *n* (%)	127 (72.2%)	94 (86.2%)	**0.006**
Hypertension, *n* (%)	166 (93.3%)	93 (85.3%)	**0.028**
Diabetes mellitus, *n* (%)	61 (34.3%)	46 (42.2%)	0.177
Dyslipidemia, *n* (%)	153 (86.0%)	90 (82.6%)	0.44
Weight (kg), mean ± SD	83.5 ± 15.2	86.1 ± 17.8	0.309

Note: Bold *p*-values denote *p* < 0.05. Values are mean ± SD, median [IQR], or *n* (%), with contributing *n* shown where incomplete. CRP-low ≤ 10 mg/L; CRP-high > 10 mg/L; PCT-positive ≥ 0.5 ng/mL. Abbreviations: CRP, C-reactive protein; HFrEF, heart failure with reduced ejection fraction; HFmrEF, heart failure with mildly reduced ejection fraction; HFpEF, heart failure with preserved ejection fraction; LVEF, left ventricular ejection fraction; NYHA, New York Heart Association; NT-proBNP, N-terminal pro B-type natriuretic peptide; PCT, procalcitonin; SD, standard deviation; IQR, interquartile range.

**Table 3:** Clinical profiles of the four CRP + PCT concordance phenotypes (Group A, CRP−/PCT−; Group B, CRP−/PCT+; Group C, CRP+/PCT−; Group D, CRP+/PCT+; *n* = 147).

Variable	A: CRP−/PCT− (*n* = 63)	B: CRP−/PCT+ (*n* = 6)	C: CRP+/PCT− (*n* = 61)	D: CRP+/PCT+ (*n* = 17)
Age (yrs), mean ± SD	71.5 ± 9.7	77.0 ± 6.2	71.9 ± 12.9	73.0 ± 9.0
LVEF (%), mean ± SD	48.5 ± 12.4	51.0 ± 16.1	49.5 ± 15.0	53.5 ± 10.8
NT-proBNP (pg/mL), median [IQR]	1778.5 [351.0–6586.0] (*n* = 54)	3714.5 [538.0–12,883.0] (*n* = 6)	3621.5 [1003.0–5838.0] (*n* = 50)	6511.5 [2205.0–18,993.0] (*n* = 12)
CRP (mg/L), median [IQR]	3.2 [1.5–7.1] (*n* = 63)	5.3 [2.4–8.2] (*n* = 6)	17.2 [15.2–23.2] (*n* = 61)	22.5 [14.8–26.1] (*n* = 17)
PCT (ng/mL), median [IQR]	0.0 [0.0–0.0] (*n* = 63)	1.3 [0.5–5.6] (*n* = 6)	0.0 [0.0–0.1] (*n* = 61)	1.6 [0.9–5.1] (*n* = 17)
NYHA III–IV, *n* (%)	27 (42.9%)	2 (33.3%)	29 (47.5%)	9 (52.9%)
HFrEF, *n* (%)	13 (20.6%)	1 (16.7%)	15 (24.6%)	2 (11.8%)
HFpEF, *n* (%)	28 (44.4%)	3 (50.0%)	32 (52.5%)	12 (70.6%)
Furosemide, *n* (%)	49 (80.3%)	4 (66.7%)	54 (88.5%)	14 (82.4%)
Leukocytes (×10^9^/L), median [IQR]	8.5 [7.3–10.2] (*n* = 63)	10.8 [6.1–16.0] (*n* = 6)	10.6 [7.6–13.2] (*n* = 61)	15.6 [8.3–16.8] (*n* = 17)

Note: Groups are biomarker-defined (CRP > 10 mg/L; PCT ≥ 0.5 ng/mL) and are not equated with confirmed clinical infection. Values are mean ± SD or median [IQR] with contributing *n*. Abbreviations: CRP, C-reactive protein; PCT, procalcitonin; LVEF, left ventricular ejection fraction; NT-proBNP, N-terminal pro-B-type natriuretic peptide; IQR, interquartile range; SD, standard deviation; NYHA, New York Heart Association; HFrEF, heart failure with reduced ejection fraction; HFpEF, heart failure with preserved ejection fraction.

**Table 4:** Baseline characteristics of NT-proBNP responders (≥30% reduction from admission to discharge) versus non-responders (*n* = 42).

Variable	NT-proBNP ≥ 30% Reduction (*n* = 29, 69%)	NT-proBNP < 30% Reduction (*n* = 13, 31%)	*p*-Value
Age (yrs), mean ± SD	74.9 ± 10.5	71.8 ± 12.0	0.488
LVEF (%), mean ± SD	49.2 ± 13.8	43.7 ± 17.0	0.237
NT-proBNP at admission (pg/mL), median [IQR]	6586.0 [3176.0–14,616.0]	3293.0 [809.0–5838.0]	**0.031**
CRP at admission (mg/L), median [IQR]	13.8 [5.3–17.2]	9.3 [5.6–22.4]	0.924
PCT at admission (ng/mL), median [IQR]	0.0 [0.0–0.2]	0.0 [0.0–0.1]	0.65
Body weight (kg), mean ± SD	84.6 ± 17.3	91.9 ± 24.0	0.454
Male sex, *n* (%)	12 (41.4%)	7 (53.8%)	0.453
HFrEF (<40%), *n* (%)	7 (24.1%)	5 (38.5%)	0.342
HFmrEF (40–49%), *n* (%)	6 (20.7%)	4 (30.8%)	0.478
HFpEF (≥50%), *n* (%)	16 (55.2%)	4 (30.8%)	0.143
NYHA III–IV at admission, *n* (%)	17 (58.6%)	9 (69.2%)	0.513
CRP > 10 mg/L, *n* (%)	16 (55.2%)	6 (46.2%)	0.588
PCT ≥ 0.5 ng/mL, *n* (%)	5 (20.8%)	0 (0.0%)	0.118
Furosemide at admission, *n* (%)	27 (93.1%)	11 (84.6%)	0.386
Diabetes mellitus, *n* (%)	11 (37.9%)	6 (46.2%)	0.616
Hypertension, *n* (%)	25 (86.2%)	13 (100.0%)	0.159
CRP > 10 mg/L, OR [95% CI]	OR = 1.44 [95% CI 0.39–5.34]		

Note: Bold *p*-values denote *p* < 0.05. Responders achieved ≥30% NT-proBNP reduction from admission to discharge. Values are mean ± SD, median [IQR], or *n* (%). Abbreviations: NT-proBNP, N-terminal pro-B-type natriuretic peptide; LVEF, left ventricular ejection fraction; CRP, C-reactive protein; PCT, procalcitonin; SD, standard deviation; IQR, interquartile range; NYHA, New York Heart Association; HFrEF, heart failure with reduced ejection fraction; HFmrEF, heart failure with mildly reduced ejection fraction; HFpEF, heart failure with preserved ejection fraction; OR, odds ratio; CI, confidence interval.

## Data Availability

The raw data supporting the conclusions of this article will be made available by the authors on request.

## Supplementary Materials

The following supporting information can be downloaded at: https://www.mdpi.com/article/10.3390/biomedicines14081795/s1, Table S1: Comparison of patients with versus without each optional biomarker measurement (PCT, serial CRP, and paired NT-proBNP), by age, admission biomarkers, and symptom severity.

## Abbreviations

The following abbreviations are used in this manuscript:

AHF	acute heart failure
APC	article processing charge
CI	confidence interval
CRP	C-reactive protein
ECLIA	electrochemiluminescence immunoassay
ESC	European Society of Cardiology
HF	heart failure
HFmrEF	heart failure with mildly reduced ejection fraction
HFpEF	heart failure with preserved ejection fraction
HFrEF	heart failure with reduced ejection fraction
IL-6	interleukin-6
IQR	interquartile range
LVEF	left-ventricular ejection fraction
NT-proBNP	N-terminal pro-brain natriuretic peptide
NYHA	New York Heart Association
OR	odds ratio
PCT	procalcitonin
SD	standard deviation
STROBE	Strengthening the Reporting of Observational Studies in Epidemiology
ΔCRP	intra-hospital change in C-reactive protein (discharge CRP minus admission CRP)
ACEi	angiotensin-converting-enzyme inhibitor
ARB	angiotensin-receptor blocker
ARNI	angiotensin-receptor–neprilysin inhibitor
GDMT	guideline-directed medical therapy
MRA	mineralocorticoid-receptor antagonist
SGLT2	sodium-glucose cotransporter-2
WBC	white blood cell (leukocyte) count
