# Supplementary material for: Inflammatory Phenotypes and Biomarker Trajectories During Hospitalization for Acute Heart Failure
Source: Biomedicines. 2026 Aug 10;14(8):1795. doi: 10.3390/biomedicines14081795 (PMC13509743; doi:10.3390/biomedicines14081795)
Supplement: Supplementary file 1 [file biomedicines-14-01795-s001.zip › biomedicines-4461080-supplementary.pdf]

Supplementary Materials

Inflammatory Phenotypes and Biomarker Trajectories During Hospitalization for Acute Heart Failure

Raluca Ibănescu, Sebastian Ciurescu, Roxana Buzaș, et al.

Table S1. Comparison of patients with versus without each optional biomarker measurement.

Patients selected for optional testing were older and more severely ill, indicating that the trajectory and NT-proBNP-response analyses derive from a higher-acuity subset and should be read as exploratory.

**Measurement / characteristic**	**Measured**	**Not measured**	**P**
**PCT measured (n = 149) vs not (n = 157)**			
Age, years (mean)	72.2	69.3	**0.011**
Admission CRP, mg/L (median)	11.6	3.7	**<0.001**
Admission NT-proBNP, pg/mL (median)	3060	1045	**<0.001**
NYHA III–IV, %	45.6	26.8	**0.001**
**Serial CRP measured (n = 65) vs not (n = 241)**			
Age, years (mean)	74.3	69.7	**0.006**
Admission CRP, mg/L (median)	14.6	4.4	**<0.001**
Admission NT-proBNP, pg/mL (median)	4362	1160	**<0.001**
NYHA III–IV, %	50.8	32.0	**0.005**
**Paired NT-proBNP measured (n = 42) vs not (n = 264)**			
Age, years (mean)	74.0	70.2	**0.034**
Admission CRP, mg/L (median)	13.4	6.1	**0.004**
Admission NT-proBNP, pg/mL (median)	5142	1267	**<0.001**
NYHA III–IV, %	61.9	31.8	**<0.001**

Sex, LVEF, and the proportion with HFpEF did not differ significantly across any comparison. P-values from the Mann–Whitney U test (continuous) or the chi-square/Fisher exact test (categorical). CRP, C-reactive protein; PCT, procalcitonin; NT-proBNP, N-terminal pro-B-type natriuretic peptide; NYHA, New York Heart Association; LVEF, left ventricular ejection fraction; HFpEF, heart failure with preserved ejection fraction.
